# Noncoding RNA and Cardiomyocyte Proliferation

**DOI:** 10.1155/2017/6825427

**Published:** 2017-10-31

**Authors:** Shuang Qu, Chunyu Zeng, Wei Eric Wang

**Affiliations:** Department of Cardiology, Chongqing Institute of Cardiology & Chongqing Cardiovascular Clinical Research Center, Daping Hospital, Third Military Medical University, Chongqing 400042, China

## Abstract

It is acknowledged that postnatal mammalian cardiomyocytes (CMs) turn over with a very limited efficacy in both physiological and pathological conditions. Recent studies showed that those newly formed CMs are derived from preexisting CMs. Thus, stimulating CM proliferation becomes a promising strategy for inducing cardiac regeneration. Noncoding RNAs were found differently expressed in CMs with different proliferation potential. Moreover, manipulation of noncoding RNAs, in particular microRNAs, was proved to promote or suppress CM proliferation, indicating that noncoding RNAs are involved in the underlying mechanism of CM proliferation. This review mainly summarizes the roles of noncoding RNAs, as a class of influential factors, in the regulation of CM proliferation.

## 1. Introduction

Cardiovascular diseases are the leading causes of morbidity and mortality all over the world. Particularly, myocardial infarction (MI) and heart failure following myocardial ischemia can lead to a large number of CM death [1]. In the past, the adult mammalian CMs are regarded as terminally differentiated cells without the ability to proliferate. Fetal CMs proliferate during development but lose this ability quickly after birth, and myocardium goes through a hyperplastic to hypertrophic transition. After this transition, the predominant form of growth is an increase in cell size and myofibril density rather than the number of CMs [2].

It is now recognized that a low level of postnatal CM proliferation was demonstrated in both normal and injured hearts. Taking advantage of integration of ^14^C into DNA to establish the age of CMs in human, a seminal study carried by Bergmann and his colleagues indicated that about 0.5–1% of CMs renews every year, so nearly 50% of CMs is replenished over a life span [3]. Recently, a combination of genetic fate mapping with stable isotope labeling and multi-isotope imaging mass spectrometry shows the renewal of CMs is predominantly from the division of preexisting CMs, rather than the differentiation from the stem cells or progenitors [4]. Our previous study showed that mature adult CMs can reenter the cell cycle and form new CMs through a three-step process, dedifferentiation, proliferation, and redifferentiation [5]. However, the proliferation is not enough to replenish the lost CMs and repair the injured myocardium, and the underlying mechanism regulating CM proliferation is still unclear. To decipher the molecular mechanism controlling CM proliferation is of great importance for stimulating the endogenous cardiac regeneration, which might be a new therapeutic approach to those patients suffering from heart diseases.

Noncoding RNAs are those RNAs which cannot code proteins, such as microRNAs (miRNAs), long noncoding RNAs (lncRNAs), and circular RNAs, and were found to play important roles in the regulation of multiple cellular activities including proliferation [6]. This review mainly summarizes the roles of noncoding RNAs in the regulation of mammalian CM proliferation.

## 2. The Role of miRNAs in CM Proliferation

MicroRNA is a small noncoding RNA molecule containing 20 ~ 24 nucleotides. Each miRNA can have multiple target genes, and it can have various spatial and temporal expression patterns which express differently in diverse tissues and developmental stages [7]. An miRNA array showed that, among the over 1000 miRNAs analyzed, 204 miRNAs increased and 311 miRNAs decreased during neonatal rat CM proliferation [8]. miRNAs were demonstrated to influence CM proliferation in neonatal and adult stages, which were summarized in Table 1.

## 3. miRNAs Regulate Neonatal CM Proliferation

The proliferation capacity of mammalian CM is robust in fetal period and is switched off early after birth. In mouse, the 1-day-old neonatal hearts can regenerate after partial surgical resection, but this capacity is lost by 7 days of age [9].

MiR-499 is a miRNA which is abundantly found in CMs and almost does not express in human cardiac stem cells or human embryonic stem cells [10]. By transfecting with pre-miR-499, EdU incorporation indicated CM proliferation was increased by 50% [11]. MiR-499 displayed a highlighted ability to promote neonatal CM proliferation via its function on Sox6 and cyclin D1 [12]. Sox6 played a role in cell viability, inhibited cell proliferation, and promoted cell apoptosis [13]. MiR-410 and miR-495 both belong to Gtl2-Dio3 miRNAs and were reported to promote CM proliferation. Overexpressing miR-410 and miR-495 in NRVMs induced about a 2.5-fold increase of proliferation analyzed with EdU incorporation assay. Meanwhile, Ki-67 immunostaining showed a threefold increase of proliferation [14]. The target gene of miR-410 and miR-495 is Cited2, a coactivator required for proper cardiac development. Cited2 knockdown reduced the expression of cell cycle inhibitor Cdkn1c/p57/Kip2 in neonatal CMs [14]. In the ischemic injury model, miR-222-overexpressing mice showed a twofold phosphohistone 3 (PH3) CMs compared with controls. Inhibition of miR-222 *in vivo* blocked CM proliferation in response to exercise. Cell cycle inhibitor P27, HIPK-1, and HIPK-2 as well as HMBOX1 were found to be involved in miR-222-induced CM proliferation [15].

MiR-133a knockdown mice hearts showed excessive CM proliferation, while miR-133a overexpression transgenic mice showed a diminished CM proliferation, indicating that miR-133 could be an inhibitor of CM proliferation [16, 17]. Similarly, miR-29a also suppressed CM proliferation, while inhibiting miR-29a promoted CM division [18]. Inhibiting miR-29a in neonatal CMs promoted CM proliferation by threefold analyzed by Ki-67 and PH3 staining and decreased the number of CMs in G0/G1 phases, while increased proportion of CMs in S and G2/M phases, indicating that inhibition of miR-29a facilitates the transition of G1/S and G2/M in CMs [19].

## 4. miRNAs Regulate Adult CM Proliferation

Hsa-miR-590-3p and hsa-miR-199a-3p are found to induce the proliferation of not only neonatal CMs but adult CMs. By injecting synthetic miRNAs directly into the heart of neonatal mice, EdU incorporation analysis revealed a marked increase of CM proliferation. Injection of AAV9 vector-expressing hsa-miR-590 or hsa-miR-199a precursor miRNAs increased CM proliferation in both neonatal and adult mice [8]. These two miRNAs can also stimulate CM proliferation in post-MI heart, which contributes to the preserved cardiac function [8].

Overexpressing miR-204 improved CM proliferation in neonatal and adult mice CMs *in vitro*. Knockdown of its target gene Jarid2 had a similar effect as miR-204 overexpression. Transgenic mice with cardiac-specific overexpression of miR-204 showed an increase of CM proliferation throughout the embryonic and adult stages, which was associated with upregulated cell cycle regulators Cyclin A, Cyclin B, Cyclin D2, Cyclin E, CDC2, and PCNA [20].

MiR-17-92, an oncogenic miRNA cluster, proved to be essential for CM proliferation and participated in the regulation of CM proliferation in embryonic, postnatal, and adult hearts. CM proliferation decreased by about 50% in postnatal hearts of miR-17–92 cKO mice, while it was significantly increased in cardiac-specific miR-17-92 overexpressed transgenic mice analyzed with PH3 and Aurora B immunostaining [21]. Overexpression of MiR-17-3p, a member of miR-17-92 cluster, has been shown to promote CM proliferation in neonatal CMs. Furthermore, inhibition of miR-17-3p attenuated exercise-induced cardiac growth and CM proliferation in adult mouse heart [22].

Loss of miR302-367 led to decreased CM proliferation during development, while increased miR302-367 expression led to a profound increase in CM proliferation. Reexpression of miR302-367 by using miRNA mimic-based treatment promoted adult CM proliferation and reduced scar formation in the post-MI heart. The CM proliferation was evaluated with Ki-67, PH3, and Aurora B kinase staining, as well as CM number counting [23]. Besides, miR-302-367 can not only have an effect on cell cycle activity but also the nucleation of CMs evidenced by an increase of the proportion of mononucleated/binucleated CMs versus multinucleated CMs in miR302-367 gain of function mice. With the method of high-throughput sequencing of RNA isolated by cross-linking immunoprecipitation (HITS-CLIP), the miR302-307 target genes Mst1, Lats2, and Mob1b are found to be components of Hippo signaling pathway, indicating the effect of miR302-367 on CM proliferation might be through repression of the Hippo signal transduction pathway [24].

MiR-34a expressed at a low level in fetal and early postnatal hearts; it soon expressed relatively higher after the first week after birth and sustained during adulthood [25]. Cardiac injury further upregulated the expression of miR-34a [26]. In the early postnatal mice, overexpression of miR-34a can decrease the CM proliferation. In contrary, antagonism of miR-34a promoted the CM proliferation through targeting on Bcl2, Cyclin D1, and Sirt 1 in the adult mice with MI injury [25]. Overexpression of miR-195, a member of the miR-15 family, during development caused premature CM cell cycle arrest, leading to congenital heart hypoplasia [27]. MiR-195 was found to be upregulated by sixfold at postnatal day 10 compared to postnatal day 1 [28]; miR-195 overexpression prevented cardiac regeneration of postnatal day 1 hearts suffering from MI injury [27]. Overexpressing miR-195 increased the proportion of NRVMs in G2/M phase [28]. In adult MI model, inhibition of miR-15 family by using administrating-locked nucleic acid- (LNA-) modified anti-miRNAs resulted in an increase in CM proliferation, with a fivefold increase of PH3-positive CMs [27]. Consistently, a chemically modified RNA oligonucleotide blocking the seed sequence of the miR-15 family members promoted adult CM proliferation and preserved cardiac function after injury [18]. In mice with miR-1-2 deletion, CM proliferation was increased, supported by 20% increased CM numbers and threefold more PH3 positive CMs [29].

## 5. Cell Cycle and Methodology for Evaluating CM Proliferation

**Figure 1:**
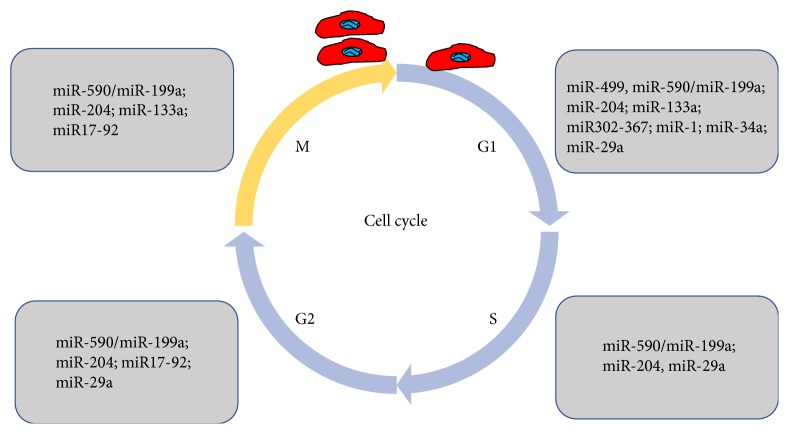
A summary of miRNAs in regulating cardiomyocyte proliferation at different phases of cell cycle.

Cell cycle can be divided into four phases: G1 phase, S phase (synthesis), G2 phase (collectively known as interphase), and mitotic (M) phase. Mitosis (division of the nucleus) and cytokinesis (division of cytoplasm, organelles, and cell membrane) together define the M phase of CM cell cycle [30]. The process of mitosis is divided into five stages: prophase, prometaphase, metaphase, anaphase, and telophase. It leads to multinucleated CMs if there is karyokinesis without cytokinesis during M-stage, and it leads to polyploid if there is DNA replication without karyokinesis and cytokinesis [31]. When considering the intrinsic proliferative capacity of adult mammalian CMs, it is important to reiterate that DNA synthesis does not necessarily result in genome duplication, that genome duplication does not necessarily result in mitosis, and that mitosis does not necessarily result in cytokinesis [32]. miRNAs regulate CM proliferation at different phases of cell cycle, which were summarized in Figure 1.

In most studies, the methods used for quantifying CM proliferation were based on immunostaining for DNA synthesis and cell cycle activation (BrdU/EdU incorporation, Ki-67, PH3, Aurora B, and alinin). BrdU/EdU can be incorporated into newly synthesized DNA at S phase of cell cycle. Ki-67 is detected in all active stages of cell cycle, including G1, S, G2, and M phases and is not active in the resting G0-phase cells and terminally differentiated cells [33]. K-i67 could not clearly distinguish multinucleated cells and polyploid with proliferating cells. Aurora B kinase is a protein that functions in the attachment of the mitotic spindle to the centromere, which is expressed during metaphase, anaphase and, cytokinesis in CMs [34]. In metaphase, Aurora B is associated to the chromosomes, whereas in anaphase and telophase it is localized to the midzone and midbody, respectively [34]. PH3 expresses during mitosis since chromosome condensation at mitosis is accompanied not only with phosphorylation of histone H3. Actually, all these markers could not identify a CM completing the whole cell cycle process and giving rise to two/multiple daughter cells. CMs especially adult ones are able to reenter into cell cycle but difficult to pass cytokinesis phase. However, none of these markers directly examine CM proliferation with cytokinesis. On the other hand, these methods could be complicated by DNA repair, polyploidy, and multinucleation in CMs. One must keep the interpretive restrictions in mind when comparing the results from different laboratories particularly when different assays are utilized.

Therefore, rigorous confirmation with nonambiguous molecular genetic markers or method should be requisite for any studies assessing de novo cardiomyogenesis. Time-lapse imaging observation combined with nuclei staining is a direct way to assess the CMs' proliferation and visualize the mitosis and cytokinesis processes [5]. This method could be an appropriate *in vitro* experiment, but new techniques are needed to quantify the CM proliferation *in vivo*.

## 6. Discussion

Understanding the underlying mechanism regulating CM proliferation could be of great clinical significance for treating MI, heart failure, and other cardiac diseases in which reduced CM numbers are the principal reason for deranged cardiac function. Noncoding RNAs especially miRNAs were demonstrated to play essential roles in CM proliferation in fetal and neonatal as well as adult stages. Manipulating the key miRNAs could be a promising strategy for stimulating cardiac regeneration in injured hearts. Other noncoding RNAs such as lncRNAs (with more than 200 nucleotides in length) may also play a role in CM proliferation. LncRNA expressions significantly changed in cardiac hyperplastic to hypertrophic growth transition [35]. Manipulation of lncRNA-Gas5 and Sghrt in adult heart reduced the expression of cell cycle regulating genes including Ccng1 and Ccnd2 in CMs [36]. The effect of these noncoding RNAs including lncRNAs and circular RNAs on CM proliferation requires further study.

## Figures and Tables

**Table 1:** A summary of the effect of miRNAs on neonatal and adult cardiomyocyte proliferation analyzed with different methodologies.

MicroRNAs	miR-499	miR-410/mi-R495	miR-590/miR-199a	miR-204	miR-195 (miR15 family)	miR-34a	miR17-92 cluster	miR-17-3p	miR-222	miR302-367	miR-29a	miR-133	miR-1
Species	Mice	Mice	Mice	Mice	Mice	Mice	Mice	Mice	Mice	Mice	Mice	Mice and zebrafish	Mice
Experiment	*In vitro*: neonatal CMs Mouse P19CL6 cells	*In vitro*: neonatal cms	*In vivo*: neonatal adult	*In vitro*: neonatal cms and adult cms *In vivo*: adult miRNA-204 transgenic mice	*In vivo:* transgenic mice	*In vitro*: neonatal cms *In vivo*: neonatal mi and adult mi	*In vivo*: transgenic mice	*In vitro*: NRVMs *In vivo*: adult myocardial ischemia-reperfusion injury	*In vitro*: NRVMs *In vivo:* exercise model	*In vitro*: transgenic mice and adult mice treated with miRNA mimics for mir302-367	*In vitro*: neonatal cms	*In vivo*: transgenic mice	*In vivo*: transgenic mice
Methods and fold changes	EdU (~1.5-fold)	EdU (~2.5-fold) Ki-67 (~3 fold)	EdU (~3-fold)	EdU (NRVMs) ~8-fold Aurora B (NRWMs) ~2-fold NRVMs numbers:~1.2-fold ARVM numbers: ~1.2-fold PH3 (transgenic mice): ~3-fold	PH3: ~2-fold	PH3: neonatal MI ~3-fold Adult MI ~8-fold EdU: NRVMs ~3-fold	Adult: CM numbers ~3-fold PH3 ~3-fold	EdU: ~1.5-fold K-i67: ~2.3-fold Cell number: ~1.2-fold	EdU: ~4-fold Ki-67:~3-fold Cell number: ~1.2-fold	Adult: CM number ~1.6-fold Neonatal: pH 3 ~6-fold Embryonic: pH 3 ~4-fold	Ki-67(~3-fold) PH3 (~3-fold)	PH3: ~2-fold	PH3: ~4-fold CM numbers: ~1.2-fold
Effect	Promote	Promote	Promote	Promote	Suppress	Suppress	Promote	Promote	Promote	Promote	Suppress	Suppress	Suppress
Target genes	Sox6 and Cyclin D1	Cited2	HOMER1, HOPX, and CLIC5	Jarid2	Check1	PNUTs, SRT1, Bcl2, Cyclin D1, and Sirt1	PTEN	TIMP-3 PTEN	P27, HMBOX1, HIPK-1, and HIK-2	Mst1, Lats2, and Mob1b	Cyclin D2, Akt3, and CDK2	CRF and Cyclin D2	Hand2 and SRF
Stage	Neonatal	Neonatal	Neonatal and adult	Neonatal and adult	Neonatal and adult	Neonatal and adult	Embryonic and neonatal adult	Neonatal	Neonatal	Embryonic and neonatal Adult	Neonatal	Neonatal	Adult
Reference	[11, 12]	[14]	[8]	[20]	[27, 28]	[26]	[21]	[22]	[15]	[23, 24]	[19]	[16, 17]	[29]
